# Aumento de Captação Cardíaca de ^18^F-FDG Induzida por Quimioterapia em Pacientes com Linfoma: Um Marcador Precoce de Cardiotoxicidade?

**DOI:** 10.36660/abc.20210463

**Published:** 2022-04-25

**Authors:** Mayara L. C. Dourado, Luca T. Dompieri, Glauber M. Leitão, Felipe A. Mourato, Renata G. G. Santos, Paulo J Almeida, Brivaldo Markman, Marcelo D. T. Melo, Simone C. S. Brandão

**Affiliations:** 1 Departamento de Pós-Graduação em Ciências da Saúde Universidade Federal de Pernambuco Recife PE Brasil Departamento de Pós-Graduação em Ciências da Saúde, Universidade Federal de Pernambuco, Recife, PE – Brasil; 2 Faculdade de Medicina Universidade Federal de Pernambuco Recife PE Brasil Faculdade de Medicina, Universidade Federal de Pernambuco, Recife, PE – Brasil; 3 Hospital das Clínicas Universidade Federal de Pernambuco Recife PE Brasil Serviço de Oncologia, Hospital das Clínicas/Universidade Federal de Pernambuco, Recife, PE – Brasil; 4 Real Hospital Português Recife PE Brasil Real Nuclear, Real Hospital Português, Recife, PE – Brasil; 5 Departamento de Medicina Interna Universidade Federal da Paraíba João Pessoa PB Brasil Departamento de Medicina Interna, Universidade Federal da Paraíba, João Pessoa, PB – Brasil

**Keywords:** Cardiotoxicidade, Quimioterapia, Linfoma

## Abstract

**Fundamento:**

Ainda não está estabelecido se a captação de fluorodesoxiglicose no miocárdio ocorre exclusivamente por características fisiológicas ou se representa um desarranjo metabólico causado pela quimioterapia.

**Objetivo:**

Investigar os efeitos da quimioterapia no coração dos pacientes com linfoma por tomografia por emissão de pósitrons associada a tomografia computadorizada (PET/CT) com 2-[18F]-fluoro-2-desoxi-D-glicose (^18^F-FDG PET/CT) antes, durante e/ou após a quimioterapia.

**Métodos:**

Setenta pacientes com linfoma submetidos a ^18^F-FDG PET/CT foram retrospectivamente analisados. O nível de significância foi de 5%. A captação de ^18^F-FDG foi avaliada por três medidas: captação máxima no ventrículo esquerdo ( *standardized uptake value* , SUV max), razão SUV cardíaco / aorta e SUV cardíaco / SUV no fígado. Também foram comparados peso corporal, glicemia de jejum, tempo pós-injeção e dose administrada de ^18^F-FDG entre os exames.

**Resultados:**

A idade média foi de 50,4 ± 20,1 anos e 50% dos pacientes eram mulheres. A análise foi realizada em dois grupos – PET/CT basal vs. intermediário e PET/CT basal vs pós-terapia. Não houve diferença significativa entre as variáveis clínicas e do protocolo dos exames entre os diferentes momentos avaliados. Nós observamos um aumento na SUV máxima no ventrículo esquerdo de 3,5±1,9 (basal) para 5,6±4,0 (intermediário), p=0,01, e de 4,0±2,2 (basal) para 6,1±4,2 (pós-terapia), p<0,001. Uma porcentagem de aumento ≥30% na SUV máxima no ventrículo esquerdo ocorreu em mais da metade da amostra. O aumento da SUV cardíaca foi acompanhado por um aumento na razão SUV máxima no ventrículo esquerdo / SUV máxima na aorta e SUV média no ventrículo esquerdo /SUV média no fígado.

**Conclusão:**

O estudo mostrou um aumento evidente na captação cardíaca de ^18^F-FDG em pacientes com linfoma, durante e após quimioterapia. A literatura corrobora com esses achados e sugere que a ^18^F-FDG PET/CT pode ser um exame de imagem sensível e confiável para detectar sinais metabólicos precoces de cardiotoxicidade.

## Introdução

A cardiotoxicidade (CTX) induzida por quimioterapia e radioterapia abrange várias formas de lesão ao sistema cardiovascular que induz uma produção aumentada de espécies reativas de oxigênio (EROs) e de nitrogênio, peroxidação lipídica e inflamação. Tal quadro leva à apoptose de cardiomiócitos e à fibrose intersticial, aumentando o risco de disfunção endotelial coronariana, disfunção ventricular esquerda e insuficiência cardíaca.^1 - 3^

Atualmente a CTX é monitorada por ecocardiografia periódica para avaliação de fração de ejeção do ventrículo esquerdo (FEVE) reduzida e/ou strain longitudinal global reduzido.^4^ No entanto, o diagnóstico de CTX baseado nesses parâmetros de função cardíaca é feito tardiamente, e pode ser indicativo de uma lesão importante e irreversível do miocárdio.^5 , 6^ Assim, é necessário examinar anormalidades do miocárdio a nível subcelular para uma avaliação sensível e precoce de CTX induzida por medicamentos.^7 , 8^

A medicina nuclear tem se mostrado muito útil para identificar doença subclínica induzida por tratamento do câncer.^9 - 11^ A tomografia por emissão de pósitrons associada a tomografia computadorizada (PET/CT) com 2-[18F]-fluoro-2-desoxi-D-glicose (^18^F-FDG) é amplamente utilizada na oncologia, principalmente em pacientes com linfoma.^12 , 13^ A captação e distribuição tecidual de ^18^F-FDG é variável, e dependente de vários fatores, tais como glicemia, período de jejum e medicamentos.^14^ Além disso, dados recentes sugerem que o acúmulo miocárdico de ^18^F-FDG não se deve totalmente ao consumo de glicose.^15^ A retenção do marcador mostrou-se dependente da atividade da enzima hexose-6-fosfato desidrogenase (H6PD) no retículo endoplasmático (RE).^15^ Essa enzima pode processar muitas hexoses, incluindo FDG,^16^ e desencadear uma via de fosfato pentose, preservando níveis de NADPH em resposta a estados de estresse oxidativo, como a CTX.^17^

Este estudo teve como objetivo identificar potenciais sinais metabólicos precoces de lesão cardíaca pela avaliação da captação miocárdica de ^18^F-FDG por PET/CT em pacientes com linfoma antes, durante e/ou após quimioterapia.

## Materiais e métodos

### Pacientes

Setenta pacientes diagnosticados com linfoma, submetidos a ^18^F-FDG PET/CT no serviço de medicina nuclear do Real Hospital Português em Recife, Pernambuco, Brasil, entre 01 de janeiro de 2012 e 28 de agosto de 2017, foram analisados retrospectivamente. O estudo foi aprovado pelo comitê de ética do Centro de Ciências de Saúde da Universidade Federal de Pernambuco, o qual isentou os autores de apresentação de consentimento por escrito dada a natureza retrospectiva do estudo.

Os critérios de inclusão foram: diagnóstico primário de linfoma, idade igual ou superior a 10 anos, e realização de pelo menos dois exames de ^18^F-FDG PET/CT antes, durante e/ou após a quimioterapia. Os critérios de exclusão foram ausência de PET/CT basais ou de controle, dados clínicos e de imagens indisponíveis ou sem possibilidade de serem avaliados, e terapia insulínica no dia do exame.

História e características clínicas dos pacientes, e variáveis relacionadas ao protocolo de ^18^F-FDG PET/CT registradas nos prontuários médicos foram coletadas, tais como peso, dose de ^18^F-FDG injetada, glicemia de jejum, e tempo para início da aquisição das imagens após injeção do FDG. Para os exames de imagem, quantificou-se a captação de ^18^F-FDG medindo-se os valores médio e máximo no ventrículo esquerdo (VE), na aorta (pool sanguineo) e no fígado.

Quatro pacientes tinham somente exames PET/CT basal e intermediário antes e durante quimioterapia, respectivamente, 40 somente exame basal e pós-terapia, e 26 apresentavam os três (basal, intermediário e pós-terapia). Para a análise, os pacientes foram então divididos em dois grupos, grupo 1, pacientes com dados de PET/CT basal e intermediário (n=30), e grupo 2, pacientes com dados basais e pós-terapia (n=66). Assim, alguns pacientes participaram de ambas as análises.

Cada grupo foi em seguida dividido em dois subgrupos de acordo com a mudança na SUV máxima de ^18^F-FDG no ventrículo esquerdo entre o exame basal e o exame de controle: uma porcentagem de aumento igual ou superior a 30% (grupo ≥30%), e uma mudança de captação de ^18^F-FDG inferior a 30% (grupo <30%). A escolha de um ponto de corte de 30% baseou-se no PERCIST^18^ ( *PET Response Criteria in Solid Tumors* ), um conjunto de critérios aplicados para avaliação da resposta do tumor à quimioterapia e radioterapia, por meio de mudanças metabólicas verificadas por exames de ^18^F-FDG PET/CT scans.^18^

### Protocolo 18F-FDG PET/CT

Para o exame de ^18^F-FDG PET/CT, os pacientes foram orientados a realizar jejum de pelo menos seis horas, não interromper nenhuma medicação, e não realizar exercícios físicos por 24 horas antes do exame. No dia do exame, foram medidos peso corporal (Kg) e glicemia de jejum, e ^18^F-FDG foi administrada por acesso venoso. Os níveis de glicemia deveriam ser inferiores a 180mg/dL. A ^18^F-FDG foi administrada em uma dose de atividade entre 3,7 a 4,8MBq/Kg e, após 60 minutos, as imagens foram obtidas por PET/CT (Biograph 16, Siemens Healthcare, EUA), a partir da base do crânio até o terço médio proximal do fêmur, três minutos por posição. Os parâmetros de aquisição da CT incluíram: cortes de 5mm, 120kV de voltagem, e sem administração de contraste endovenoso.

As imagens foram processadas usando reconstrução iterativa (duas iterações, oito subgrupos com filtro gaussiano) por um médico nuclear, que realizou uma análise quantitativa com SUV máxima e SUV médio. Ambos os SUVs foram medidos no ventrículo esquerdo em imagens fundidas de PET/CT e determinados de modo semiautomático, com auxílio do programa syngo versão 5.1 (Siemens Healthcare), a partir da demarcação de um volume de interesse (VOI) incluindo todo o ventrículo esquerdo. SUV máxima e médio do pool sanguíneo foram medidos pela demarcação da região de interesse (ROI) na aorta descendente logo após o arco aórtico. SUV máxima e médio do fígado foram medidos pela demarcação de uma ROI no segmento VI.

### Análise estatística

Os dados foram analisados com o programa Stata 12.1. As variáveis contínuas foram expressas em média ± desvio padrão (DP); e as variáveis categóricas em frequência e porcentagem. Comparações de porcentagem entre dois grupos independentes foram realizados pelo teste do qui-quadrado de Pearson ou teste exato de Fisher. O teste t de Student foi usado para comparar duas médias tanto de amostras independentes como de amostras pareadas. Em todos os testes, um nível de significância de 5% foi adotado para rejeitar a hipótese nula.

## Resultados

A idade média dos 70 pacientes estudados foi 50,4 ± 20,1 anos (16-88 anos), e 50% dos pacientes eram mulheres. Vinte pacientes (28,6%) apresentavam hipertensão e 10 (14,3%) diabetes. Cerca de 67% (n=47) apresentavam linfoma não-Hodgkin (LNH) e os demais, linfoma de Hodgkin (LH). Somente três pacientes (4,3%) se submeteram à radioterapia do mediastino entre o fim da quimioterapia e o exame ^18^F-FDG PET/CT de controle. Foi possível definir o regime quimioterápico em 33 pacientes (47,1%) e todos os regimes incluíram drogas cardiotóxicas ( Tabela 1 ).

**Tabela 1:** – Características clínicas e terapêuticas dos pacientes avaliados (n=70)

Variável	N (%)
**Sexo feminino**	35 (50,0)
**Hipertensão**	20 (28,6)
**Diabetes**	10 (14,3)
**Dislipidemia**	14 (20,0)
**Tabagismo**	
Não fumante	49 (70,0)
Ex-fumante	20 (28,6)
Fumante	1 (1,4)
**Consumo de álcool**	0 (0)
**Doença arterial coronariana**	5 (7,1)
**Hemodiálise**	1 (1,4)
**Medicamentos**	
Nenhum	10 (14,3)
Medicamento não cardioprotetor ^a^	40 (57,1)
Medicamento cardioprotetor ^a^	20 (28,6)
**Câncer**	
Linfoma de Hodgkin	23 (32,9)
Linfoma não Hodgkin	47 (67,1)
**Quimioterapia** ^b^	
RCHOP	11 (33,3)
RCHOP + alternativo	6 (18,2)
ABVD	11 (33,3)
ABVD + alternativo	2 (6,1)
DA-EPOCH-R	1 (3,0)
BEACOPP	1 (3,0)
RCOP	1 (3,0)
**Mediastino Radioterapia após pet basal**	**3 (4,3)**

*^a^ Medicamento cardioprotetor: bloqueador de receptor de angiotensina II, betabloqueador, inibidor da enzima conversora da angiotensina; ^b^ Disponível para 33 pacientes. ABVD: Adriamicina ou Doxorrubicina + Bleomicina + Vimblastina + Dacarbazina; BEACOPP: Bleomicina + Etoposídeo + Adriamicina ou Doxorrubicina + Ciclofosfamida + Vincristina + Procarbazina + Prednisona; DAC: Doença arterial coronariana, DA-EPOCH-R: Etoposídeo com dose ajustada + Prednisona + Vincristina + Ciclofosfamida + Doxorrubicina ou Hidroxidoxorrubicina + Rituximab, RCHOP: Rituximab + Ciclofosfamida + Doxorrubicina ou Hidroxidoxorrubicina + Vincristina + Prednisona, RCOP: Rituximab + Ciclofosfamida + Vincristina + Prednisona; PET: tomografia por emissão de pósitrons.*

### Grupo 1: 18F-FDG PET/CT basal e intermediário

Houve padronização do protocolo ^18^F-FDG PET/CT entre o exame basal e o exame intermediário. Não houve diferença na dose de ^18^F-FDG administrada, glicemia de jejum, e tempo decorrido quando comparados os exames basal e intermerdiario. O peso médio dos pacientes também não mudou significativamente, o que possibilitou a comparação da captação de ^18^F-FDG nos órgãos-alvo ( Tabela 2 ).

**Tabela 2:** – Comparação de peso corporal, glicemia de jejum, dose injetada de 2-[18F]-fluoro-2-desoxi-D-glicose (18F-FDG), e média de tempo após injeção de 18F-FDG entre os exames de tomografia por emissão de pósitrons (PET) basal e intermediário

Variável (N=30)	Basal	Intermediário	p*
	Média ± DP	Média ± DP	
**Peso** (Kg)	75,3 ± 14,3	74,7 ± 13,5	0,551
**Glicemia de jejum** (mg/dL)	92,6 ± 19,5	93,4 ± 19,9	0,816
**Dose de** ^18^F-FDG mCi	9,1 ± 2,7	9,1 ± 2,0	0,971
**Tempo pós injeção** (min)	68,8 ± 10,0	65,9 ± 9,9	0,308

*Teste t de Student.

**Figura 1:**
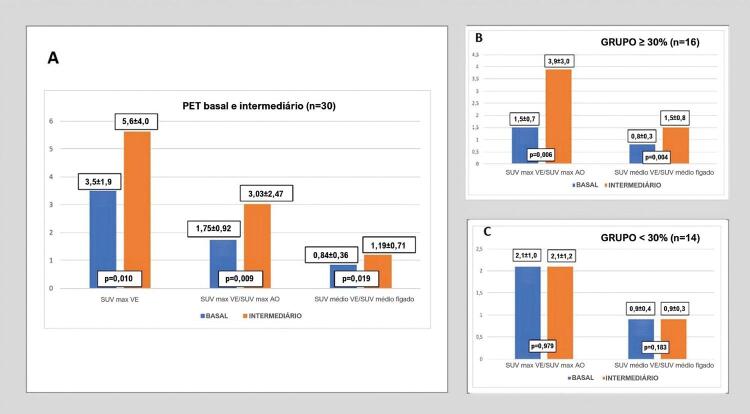
– Grupo 01 – A) Comparação do valor máximo de captação (SUV, do inglês standardized uptake value) no ventrículo esquerdo, razão SUV máxima no ventrículo esquerdo/SUV máxima na aorta e SUV médio no ventrículo esquerdo/SUV médio no fígado entre tomografia por emissão de pósitron (PET) basal e PET intermediário B) Comparação de SUV máxima no ventrículo esquerdo/SUV máxima na aorta e SUV média no ventrículo esquerdo/SUV média no fígado entre PET basal e PET intermediário no grupo com aumento na SUV máxima no ventrículo esquerdo ≥ 30% C: Comparação de SUV máxima no ventrículo esquerdo / SUV máxima na aorta e SUV média no ventrículo esquerdo/SUV média no fígado entre PET basal e PET intermediário no grupo com aumento na SUV máxima no ventrículo esquerdo < 30%; SUV max VE: SUV máxima no ventrículo esquerdo; SUV max AO: SUV máxima na aorta; SUV médio VE: SUV média no ventrículo esquerdo.

Por outro lado, a SUV máxima de ^18^F-FDG no ventrículo esquerdo aumentou no exame intermediário em comparação ao basal. Também se observou um aumento significativo na razão SUV máxima no ventrículo esquerdo/SUV máxima na aorta e na razão SUV média no ventrículo esquerdo/SUV média no fígado do exame basal ao exame intermediário ( Figura 1A ). O intervalo médio entre os exames basal e intermediário foi 95,4 ± 32,2 dias.

Dos 30 pacientes que se submeteram ao exame de ^18^F-FDG PET/CT, 16 (53,3%) apresentaram um aumento ≥30% (grupo ≥30%) na SUV máxima. Em relação às variáveis clínicas, tais como fatores de risco e medicamentos usados, não foram observadas diferenças.

Os valores de SUV máxima no ventrículo esquerdo/SUV máxima na aorta e da razão SUV média no ventrículo esquerdo/SUV média no fígado também aumentaram significativamente na avaliação intermediária em comparação à basal no grupo >= 30% ( Figura 1B ). No grupo <30% (n=14), não houve aumento estatisticamente significativo nessas razões entre o exame basal e o exame intermediário ( Figura 1C ).

### Grupo 2: 18F-FDG PET/CT basal e pós-terapia

Sessenta e seis pacientes submeteram-se a exames ^18^F-FDG PET/CT nos períodos basal e pós-terapia. Não houve diferença na glicemia de jejum, na dose de atividade de ^18^F-FDG administrada, e no tempo pós-injeção entre as duas avaliações. O peso médio dos pacientes foi ligeiramente maior no exame pós-terapia em comparação à média de peso basal ( Tabela 3 ).

**Tabela 3:** – Comparação de peso corporal, glicemia de jejum, dose injetada de 2-[18F]-fluoro-2-desoxi-D-glicose (18F-FDG), e média de tempo após injeção de 18F-FDG entre os exames de tomografia por emissão de pósitrons (PET) basal e pós-terapia

Variável (N=66)	Basal	Pós-Terapia	p*
	Média ± DP	Média ± DP	
**Peso** (Kg)	72,7 ± 14,8	75,2 ± 15,2	0,014
**Glicemia de jejum** (mg/dL)	91,6 ± 15,6	91,6 ± 16,7	>0,99
**Dose de** ^18^F-FDG mCi	9,2 ± 2,3	9,5 ± 2,2	0,308
**Tempo pós injeção** (min)	68,6 ± 9,1	70,4 ± 5,8	0,606

* Teste t de Student.

A SUV máxima no ventrículo esquerdo foi significativamente maior no PET pós-terapia. Observamos um aumento absoluto de 2,1 (IC 95% 1,3 a 3,0), o que representa uma porcentagem de aumento de 66,5% (IC 95% 43,3% a 89,7%) em relação ao exame basal.

**Figura 2:**
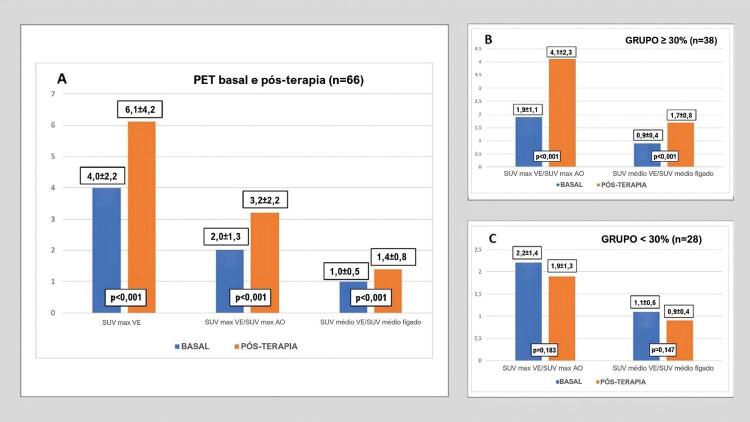
– Grupo 2: Comparação do valor máximo de captação (SUV, do inglês standardized uptake value) no ventrículo esquerdo, razão SUV máxima no ventrículo esquerdo / SUV máxima na aorta e SUV médio no ventrículo esquerdo/SUV médio no fígado entre tomografia por emissão de pósitron (PET) basal e PET pós-terapia B: Comparação de SUV máxima no ventrículo esquerdo / SUV máxima na aorta e SUV médio no ventrículo esquerdo/SUV média no fígado entre PET basal e PET pós-terapia no grupo com aumento na SUV máxima no ventrículo esquerdo ≥ 30% C: Comparação de SUV máxima no ventrículo esquerdo / SUV máxima na aorta e SUV médio no ventrículo esquerdo/SUV média no fígado entre PET basal e PET pós-terapia no grupo com aumento na SUV máxima no ventrículo esquerdo < 30%; SUV max VE: SUV máxima no ventrículo esquerdo; SUV max AO: SUV máxima na aorta; SUV médio VE: SUV médio no ventrículo esquerdo.

Os valores de SUV máxima no ventrículo esquerdo /SUV máxima na aorta e de SUV médio no ventrículo esquerdo /SUV médio no fígado também aumentaram no PET pós-terapia em comparação ao basal ( Figura 2A ). O tempo médio entre o exame basal e o exame pós-terapia foi de 231,8±125,7 dias.

Dos 66 pacientes, 38 (57,6%) apresentaram um aumento ≥ 30% na captação cardíaca de ^18^F-FDG (grupo ≥ 30%). Não houve diferenças entre os grupos quanto às variáveis clínicas, tais como fatores de risco cardiovasculares e uso de medicamentos.

Os valores de SUV máxima no ventrículo esquerdo /SUV máxima na aorta e de SUV médio no ventrículo esquerdo /SUV médio no fígado aumentaram significativamente na avaliação pós-terapia em comparação ao basal no grupo ≥ 30% ( Figura 2B ). No grupo <30% (n=28), não houve aumento significativo nessas razões ( Figura 2C ).

**Figura 3:**
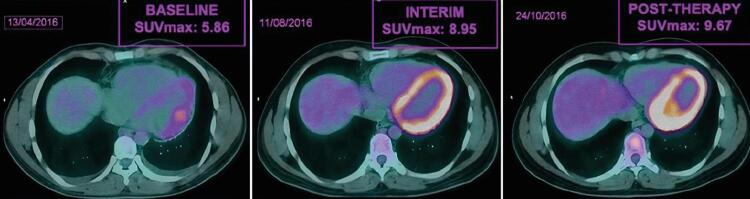
– Exemplo de caso - valor máximo de captação (SUV, do inglês standardized uptake value) (SUVmax) no ventrículo esquerdo na tomografia computadorizada por emissão de pósitron (PET/CT) basal (5,86), intermediário (8,95/52,73% de aumento a partir do basal) e pós-terapia (9,67/65,02% de aumento a partir do basal).

A Figura 3 ilustra um exemplo do comportamento da SUV máxima antes, durante e após quimioterapia.

## Discussão

O presente estudo mostrou que a quimioterapia em pacientes com linfoma causou um desequilíbrio no metabolismo cardíaco, o que foi evidenciado pela maior captação de ^18^F-FDG. Tais resultados são corroborados por evidências recentes que sugerem que essa maior captação cardíaca de FDG induzida por quimioterapia pode ser um sinal de CTX. O aumento de ^18^F-FDG cardíaco foi observado no PET intermediário e no PET pós-terapia. Tais resultados não sofreram interferência quanto à dose de atividade de ^18^F-FDG administrada ou qualquer diferença possível no preparo ou momento de realização do exame.

**Figura 4:**
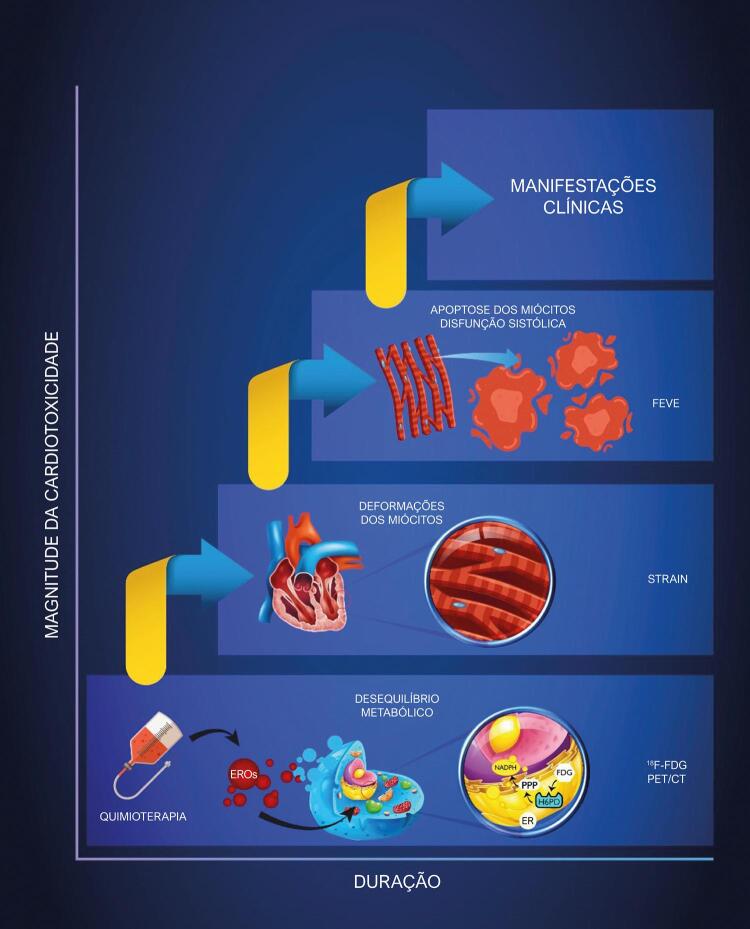
– Cascata de cardiotoxicidade – A lesão provocada por cardiotoxicidade desencadeia uma série de alterações metabólicas em resposta ao estresse oxidativo, detectáveis por tomografia computadorizada por emissão de pósitrons e 2-[18F]-fluoro-2-desoxi-D-glicose (18F-FDG) (18F-FDG PET/CT). A lesão e a falha de autorregeneração dos miócitos contribuem para a disfunção celular e alterações mecânicas detectadas pela avaliação do strain. Ainda, o processo continua com uma diminuição no desempenho cardíaco avaliado pela fração de ejeção do ventrículo esquerdo (FEVE). Sinais de insuficiência cardíaca podem ser então detectados, sugerindo que o coração já não atende as demandas corporais, ou o faz às custas de uma pressão de enchimento ventricular elevada (EROs: espécies reativas de oxigênio; RE: retículo endoplasmático; PPP: via da pentose fosfato; H6PD: hexose-6-fosfato desidrogenase; FDG: Fluorodesoxiglicose; FEVE: fração de ejeção do ventrículo esquerdo).

O exame de ^18^F-FDG PET/CT é um método bem estabelecido no diagnóstico e estadiamento de pacientes oncológicos, especialmente pacientes com linfoma, com potencial capacidade de avaliar manifestações precoces de CTX a nível subcelular, como postulado na Figura 4 .

Terapias antineoplásicas têm melhorado taxas de sobrevida em pacientes oncológicos. No entanto, seus efeitos citotóxicos mostram um amplo espectro de alterações crônicas e agudas ao sistema cardiovascular.^19^ Sabe-se que mecanismos celulares e moleculares da CTX afetam a homeostase principalmente no miocárdio e no endotélio, prejudicando significativamente a saúde cardiovascular.^20^

A CTX afeta o sistema cardiovascular primeiramente pela inibição da topoisomerase II e formação de EROs, que desencadeiam as vias apoptóticas dependente de mitocôndria (intrínseca) e dos receptores de morte celular. (extrínseca). A cascata continua com a ativação da caspase 3, expressão da fosfatidilserina, fragmentação do DNA, condensação da cromatina, e metabolização da membrana fosfolipídica.^21^ O estágio final é caracterizado pela formação de bolhas (“ *blebbing* ”) na membrana e encolhimento das células.^22^ Esse é o mecanismo subjacente da CTX subclínica que oferece várias oportunidades para avaliar sinais precoces dessa entidade.

As recomendações e diretrizes atuais baseiam-se em técnicas de imagens focadas em parâmetros anatômicos, tais como a ecocardiografia, angiografia radioisotópica (MUGA, *multigated radionuclide angiography)* , e ressonância magnética cardíaca (RMC).^23^ Contudo, essas abordagens detectam manifestações tardias da CTX com baixa sensibilidade para alterações subclínicas.^24^

As técnicas de medicina nuclear podem ser uma ferramenta para avaliar pontos específicos da via de CTX. O ^18^F-FDG PET/CT, comumente utilizado para detectar metabolismo glicolítico tumoral, tem se mostrado como um marcador útil na detecção precoce de CTX. Inicialmente, vários estudos indicaram que a doxorrubicina (DXR), uma das antraciclinas, pode afetar especificamente o metabolismo do miocárdio, como mostrado em estudo experimental.^25^

Vários estudos clínicos e experimentais têm mostrado que medicações cardiotóxicas, tais como sunitinibe e antraciclinas, aumenta a captação cardíaca de ^18^F-FDG ao longo do tempo, e está relacionada a alterações ecocardiográficas.^26 - 33^

Apesar de a captação de ^18^F-FDG ser comumente associada ao consumo de glicose, dados mais recentes mostram resultados diferentes. O estresse redox e sua resposta antioxidante têm sido caracterizadas como um possível mecanismo por trás da progressão da disfunção cardíaca na CTX e na captação de ^18^F-FDG, independentemente do metabolismo glicolítico.^34^

O estresse redox a nível do RE pode ativar a via fosfato pentose local desencadeada por H6PD para alimentar os níveis de NADPH necessários para a resposta antioxidante, e está relacionada à captação aumentada de ^18^F-FDG.^35^

Em situações de estresse oxidativo, o NADPH é uma fonte importante de elétrons para reações de redução.^36^ O NADPH é gerado no meio intraluminal pela H6PD, uma enzima bifuncional que catalisa as duas primeiras etapas da via fosfato pentose, convertendo glicose-6-fosfato em 6-fosfogluconato com produção concomitante de NADPH.^37^ H6PD tem como substrato várias hexoses como 2-desoxiglicose e ^18^F-FDG.^38^

No coração, existe uma relação direta entre o estresse oxidativo no RE e a captação de 2 - desoxiglicose,^39^ que pode ser considerada como uma fase metabólica inicial da disfunção contrátil causada pela sobrecarga de pressão.^40^ Além disso, Hrelia et al.^41^ mostraram que a captação aumentada de 2 - desoxiglicose, induzida por doxorrubicina nos cardiomiócitos pode ser revertida por um efeito antioxidante do alfa-tocoferol.^41^

Em 2019, Bauckneht et al.^33^ analisaram o efeito do dano oxidativo induzido por DXR sobre a correlação entre captação de ^18^F-FDG, consumo de glicose, e a resposta metabólica induzida por H6PD em camundongos. O estudo mostrou que o estresse redox no miocárdio persistiu e se correlacionou diretamente com o aumento na captação de ^18^F-FDG (aumento da SUV), bem como na ativação de vias antioxidantes fisiológicas, tais como a função catalítica da H6PD.^33^ O estudo também mostrou que a alteração metabólica persistiu após o desaparecimento do DXR, e precedeu a manifestação da disfunção contrátil.^33^ Estudos anteriores mostraram uma relação positiva entre a geração de EROs e a captação de ^18^F-FDG no câncer.^42^

Em conformidade com esses achados, estudos recentes mostraram um aumento na captação de ^18^F-FDG no PET/CT independente do metabolismo glicolítico, e associado à atividade enzimática de H6PD no cérebro.^43 , 44^ Outra análise mostrou a ligação entre captação de ^18^F-FDG e geração de EROs no estresse de redox induzido por hiperglicemia envolvendo ativação de H6PD.^45^

Apesar de seus resultados e fundamentos interessantes do presente estudo, sua natureza retrospectiva dificulta a avaliação dos mecanismos subjacentes à captação aumentada de ^18^F-FDG no miocárdio. Contudo, não foram identificados outros fatores cardiotóxicos, além da CTX, entre o exame basal e exame de controle na maior amostra de pacientes com linfoma avaliados durante e após a quimioterapia. Ainda, diferentemente de outros estudos, nós medimos não somente a SUV máxima no ventrículo esquerdo, como também os valores de captação no ventrículo esquerdo corrigidos pela captação hepática e pelo “pool” sanguíneo no interior da aorta, ressaltando assim o aumento da captação cardíaca do marcador durante e após quimioterapia. Ainda, o protocolo de ^18^F-FDG PET/CT e os possíveis fatores de variabilidade da SUV foram os mesmos em todos os exames basais e de controle.

Mais estudos são necessários para correlacionar a captação aumentada de ^18^F-FDG com desfechos clínicos, classe e dose de quimioterapia, níveis de troponina e NT-proBNP, e com outros métodos de imagem, tais como ecocardiografia e RMC.

## Conclusão

O presente estudo mostrou um aumento evidente na captação cardíaca de^18^F-FDG em pacientes com linfoma, evidenciado por ^18^F-FDG PET/CT durante e/ou após quimioterapia. A literatura corrobora esses achados e sugere que a captação aumentada de ^18^F-FDG possa ser um sinal precoce importante de CTX facilmente avaliado por um método amplamente disponível. Com o desenvolvimento das terapias anticâncer, a CTX ainda é uma preocupação que requer mais investigação e novas abordagens diagnósticas.
