# Supplementary material for: Aumento de Captação Cardíaca de 18F-FDG Induzida por Quimioterapia em Pacientes com Linfoma: Um Marcador Precoce de Cardiotoxicidade?
Source: Arq Bras Cardiol. 2022 Apr 25;118(6):1049–58. [Article in Portuguese] doi: 10.36660/abc.20210463 (PMC9345149; doi:10.36660/abc.20210463)
Supplement: Translation

# Translation: Chemotherapy-induced Cardiac^18^F-FDG Uptake in Patients with Lymphoma: An Early Metabolic Index of Cardiotoxicity?

**Authors:** Mayara L. C. Dourado, Luca T. Dompieri, Glauber M. Leitão, Felipe A. Mourato, Renata G. G. Santos, Paulo J Almeida, Brivaldo Markman, Marcelo D. T. Melo, Simone C. S. Brandão

**Affiliations:** 1 Brasil Departamento de Pós-Graduação em Ciências da Saúde, Universidade Federal de Pernambuco, Recife, PE – Brasil; 2 Brasil Faculdade de Medicina, Universidade Federal de Pernambuco, Recife, PE – Brasil; 3 Brasil Serviço de Oncologia, Hospital das Clínicas/Universidade Federal de Pernambuco, Recife, PE – Brasil; 4 Brasil Real Nuclear, Real Hospital Português, Recife, PE – Brasil; 5 Brasil Departamento de Medicina Interna, Universidade Federal da Paraíba, João Pessoa, PB – Brasil

**Keywords:** Cardiotoxicity, Chemotherapy, Lymphoma

## Abstract

**Background:**

It is uncertain whether myocardial fluorodeoxyglucose uptake occurs solely due to physiological features or if it represents a metabolic disarrangement under chemotherapy.

**Objective:**

To investigate the chemotherapy effects on the heart of patients with lymphoma by positron emission tomography associated with computed tomography scans (PET/CT) with 2-deoxy-2[18F] fluoro-D-glucose (^18^F-FDG PET/CT) before, during and/or after chemotherapy.

**Methods:**

Seventy patients with lymphoma submitted to^18^F-FDG PET/CT were retrospectively analyzed. The level of significance was 5%.^18^F-FDG cardiac uptake was assessed by three measurements: left ventricular maximum standardized uptake value (SUVmax), heart to blood pool (aorta) ratio, and heart to liver ratio in all the exams. Body weight, fasting blood sugar, post-injection time, and the injected dose of^18^F-FDG between the scans were also compared.

**Results:**

Mean age was 50.4 ± 20.1 years and 50% was female. The analysis was carried out in two groups: baseline vs. interim PET/CT, and baseline vs. post-therapy PET/CT. There was no significant difference in clinical variables or protocol scans variables. We observed an increase in left ventricular (LV) SUVmax from 3.5±1.9 (baseline) to 5.6±4.0 (interim), p=0.01, and from 4.0±2.2 (baseline) to 6.1±4.2 (post-therapy), p<0.001. A percentage increase ≥30% of LV SUVmax occurred in more than half of the sample. The rise of cardiac SUV was accompanied by an increase in LV SUVmax/Aorta SUVmax and LV SUVmean/Liver SUVmean ratios.

**Conclusion:**

This study showed a clear increase in cardiac^18^F-FDG uptake in patients with lymphoma during and/or after chemotherapy. The literature corroborates with these findings and suggests that^18^F-FDG PET/CT is a sensitive and reliable imaging exam to detect early metabolic signs of cardiotoxicity.

## Introduction

Chemotherapy and radiotherapy-induced cardiotoxicity (CTX) encompasses various forms of injury to the cardiovascular system, that trigger an increased production of reactive oxygen (ROS) and nitrogen species, lipid peroxidation and inflammation. This leads to cardiomyocyte apoptosis and interstitial fibrosis, increasing the risk for impaired coronary endothelial function, left ventricular (LV) dysfunction and heart failure.^1 - 3^

Today, CTX is monitored by periodic imaging with echocardiography for assessment of left ventricular ejection fraction (LVEF) reduction and/or decreased global longitudinal strain.^4^ However, the diagnosis of CTX based on these cardiac function parameters is late, and can be an indication of a significant and irreversible myocardial injury.^5 , 6^ Therefore, it is necessary to evaluate myocardial abnormalities at subcellular level for an early and sensitive assessment of drug-induced CTX.^7 , 8^

Cardiac imaging techniques of nuclear medicine have proved extremely useful to identify subclinical disease in the context of cancer therapy-induced organ damage.^9^ Positron emission tomography associated with computed tomography scans (PET/CT) with 2-deoxy-2[18F] fluoro-D-glucose (^18^F-FDG) is widely used in oncology, especially in patients with lymphoma.^12 , 13^ Tissue^18^F-FDG uptake and tissue distribution is variable and depend on several factors such as glucose level, fasting period and drugs.^14^ Furthermore, recent data suggest that myocardial^18^F-FDG accumulation is not entirely due to glucose consumption.^15^ The tracer retention was found to be dependent upon the enzymatic activity of hexose-6-phosphate-dehydrogenase (H6PD) in the endoplasmic reticulum (ER).^15^ This enzyme can process many hexoses, including FDG,^16^ to trigger a pentose phosphate pathway and preserve NADPH levels in response to oxidative stress conditions, such as CTX.^17^

This study aimed to identify potential early signs of metabolic cardiac injury by assessing changes in cardiac^18^ F-FDG uptake by PET/CT in patients with lymphoma before, during and/or after chemotherapy.

## Material and Methods

### Patients

Seventy patients diagnosed with lymphoma and submitted to^18^F-FDG PET/CT in the Division of Nuclear Medicine of Real Hospital Português in Recife, Pernambuco, Brazil, between January 1, 2012 and August 28, 2017 were retrospectively analyzed in this study. The study was approved by the Research Ethics Board of the Federal University of Pernambuco Health Sciences Center, which granted a waiver of written consent due to the retrospective nature of the study.

Inclusion criteria were primary diagnosis of lymphoma, aged 10 years or older and, at least two^18^F-FDG PET/CT scans before, during and/or after chemotherapy. Exclusion criteria were no baseline or control tests, unavailability and/or inability to assess clinical data and imaging tests, and insulin therapy on the day of the scan.

Patients’ clinical features, medical history and variables related to the^18^F-FDG PET/CT protocol recorded in their medical records were collected, such as, weight, injected dose of^18^F-FDG, fasting blood sugar (FBS) and time after injection. For imaging exams,^18^F-FDG uptake was quantified by measuring the mean and the maximum standardized uptake value (SUVmean and SUVmax, respectively).

Four patients had only baseline and interim PET/CT scans, 40 had only baseline and post-therapy and 26 had all three. For analysis, the patients were then divided into two groups, group 1, patients with baseline and interim PET/CT scan data (n = 30); and group 2, patients with baseline and post-therapy PET/CT data (n = 66). Thus, some patients participated in both analyses.

Each group was then divided in two subgroups according to the change in the LV^18^F-FDG SUVmax between baseline and control tests: a percentage increase above or equal to 30% (Group ≥ 30%), and a less than 30%^18^F-FDG uptake change (Group <30%). The choice of a 30% cutoff was based on PERCIST^18^(PET Response Criteria in Solid Tumors), which is a set of criteria for assessment of tumor response to chemotherapy and radiotherapy, through metabolic changes verified by^18^F-FDG PET/CT scans.^18^

### 18F-FDG PET/CT Protocol

For the^18^F-FDG PET/CT, patients were instructed to fast at least six hours prior to the test, not to discontinue any medication or exercise for 24 hours before the scan. On the day of the scan, body weight (kg) and FBS were measured and, venous puncture was used to administer^18^F-FDG. Blood sugar levels should be below 180 mg/dL. The^18^F-FDG was administered at an activity dose of 3.7 to 4.8MBq/kg and after 60 minutes, the images were obtained by the PET/CT (Biograph 16, Siemens Healthcare, USA), extending from the base of the skull to the proximal-middle third of the femur, three minutes per bed position. The acquisition parameters of the CT scan included: 5mm slices, 120kV voltage, and no intravenous contrast administration.

Imaging processing was done with iterative reconstruction (two iteractions, eight subsets with Gaussian filter) by a nuclear physician, who performed a quantitative analysis with SUVmax and SUVmean. Both SUVs were measured at the left ventricle on fused PET/CT images and determined semi-automatically with the aid of the syngo via software version 5.1 (Siemens Healthcare) through the demarcation of a volume of interest (VOI) including the entire left ventricle. SUVmax and SUVmean for blood pool were measured by reconstruction of a region of interest (ROI) in the descendent aorta just after the aortic arch. SUVmax and SUVmean for liver were measured by reconstruction of a ROI of 4.0 cm diameter in the VI segment.

### Statistical analysis

Data was analyzed with Stata 12.1 statistical software. Continuous variables were expressed as mean ± standard deviation (SD); and categorical variables were summarized by frequency and percentage. Percentage comparisons between two independent groups were performed using the Pearson’s chi-square test or, when it was not applicable, the Fisher’s exact test. The Student’s t-test was used to compare two means for both independent and paired samples. In all tests, a significance level of 5% was used to reject the null hypothesis.

## Results

The mean age of the 70 patients studied was 50.4 ± 20.1 years (16-88 years) and 50% were female. Twenty patients (28.6%) had hypertension and 10 (14.3%) had diabetes. About 67% (n= 47) had non-Hodgkin’s lymphoma (nHL) and the remainder (n=23) had Hodgkin’s lymphoma (HL). Only three patients (4.3%) underwent mediastinal radiotherapy between the end of chemotherapy and the control^18^F-FDG PET/CT scan. It was possible to define the chemotherapy regimen in 33 patients (47.1%) and all regimens included known cardiotoxic drugs ( Table 1 ).

**Table 1:** – Clinical and therapeutic characteristics of the patients (n=70)

Variable	N (%)
**Female sex**	35 (50.0)
**Hypertension**	20 (28.6)
**Diabetes**	10 (14.3)
**Dyslipidemia**	14 (20.0)
**Smoking**	
Non-smoker	49 (70.0)
Former smoker	20 (28.6)
Current smoker	1 (1.4)
**Alcoholism**	0 (0)
**Coronary artery disease**	5 (7.1)
**Hemodialysis**	1 (1.4)
**Medication**	
No	10 (14.3)
Non-cardioprotective medication^a^	40 (57.1)
Cardioprotective medication^a^	20 (28.6)
**Cancer**	
Hodgkin’s Lymphoma	23 (32.9)
Non-Hodgkin’s Lymphoma	47 (67.1)
**Chemotherapy** ^**b**^	
RCHOP	11 (33.3)
RCHOP + alternative	6 (18.2)
ABVD	11 (33.3)
ABVD + alternative	2 (6.1)
DA-EPOCH-R	1 (3.0)
BEACOPP	1 (3.0)
RCOP	1 (3.0)
**Mediastinal Radiotherapy After Baseline Pet**	**3 (4.3)**

*^a^Cardioprotective medication: angiotensin II receptor blocker, beta-blocker, angiotensin-converting enzyme inhibitor.^b^Available for 33 patients. ABVD: Adriamycin or Doxorubicin + Bleomycin + Vinblastine + Dacarbazine; BEACOPP: Bleomycin + Etoposide + Adriamycin or Doxorubicin + Cyclophosphamide + Vincristine + Procarbazine + Prednisolone; DA-EPOCH-R: Dose-Adjusted Etoposide + Prednisolone + Vincristine + Cyclophosphamide + Doxorubicin or Hydroxydaunorubicin + Rituximab, RCHOP: Rituximab + Cyclophosphamide + Doxorubicin or Hydroxydaunorubicin + Vincristine + Prednisolone, RCOP: Rituximab + Cyclophosphamide + Vincristine + Prednisolone.*

### Group 1: baseline and interim 18F-FDG PET/CT

There was standardization of the^18^F-FDG PET/CT protocol between the baseline and interim scans. There was no difference in the injected dose of^18^F-FDG, FBS and time post-injection between baseline and interim exams. Mean body weight of patients also did not change significantly, making it possible to compare the^18^F-FDG uptake in the target organs ( Table 2 ).

**Table 2:** – Comparison of body weight, fasting blood sugar, injected dose of 18F-fluorodeoxy glucose (18FDG), and mean post-injection time of patients between baseline and interim positron emission tomography associated with computed tomography (PET/CT) scans

Variable (N=30)	Baseline	Interim	p*
	Mean ± SD	Mean ± SD	
**Weight** (Kg)	75.3 ± 14.3	74.7 ± 13.5	0.551
**FBS** (mg/dL)	92.6 ± 19.5	93.4 ± 19.9	0.816
**Dose of** ^18^FDG mCi	9.1 ± 2.7	9.1 ± 2.0	0.971
**Post-injection time** (min)	68.8 ± 10.0	65.9 ± 9.9	0.308

*Student’s t-test. FBS: Fasting Blood Sugar

On the other hand,^18^F-FDG LV SUVmax increased at the interim scan compared to baseline. Similarly, there was a significant increase in the LV SUVmax/aorta SUVmax and LV SUVmean/liver SUVmean ratios from baseline to interim scans ( Figure 1A ). The mean time interval between baseline and interim scans was 95.4 ± 32.2 days.

**Figure 1:** – Group 01 – A) Comparison of maximum left ventricular (LV) standardized uptake value (SUVmax), LV SUVmax/aorta SUVmax and mean LV SUV (SUV mean)/liver SUVmean ratios, between baseline and interim positron emission tomography (PET). B) Comparison of LV SUVmax/aorta SUVmax and LV SUVmean/liver SUVmean ratios between baseline and interim PET in the Group with increase of LV SUVmax ≥ 30%. C) Comparison of LV SUVmax/Aorta SUVmax and LV SUVmean/Liver SUVmean ratios, between Baseline and Interim PET in the Group with increase of LV SUVmax < 30%; LVmaxAOmax: LV SUVmax/Aorta SUVmax, LVmean LIVER mean: LV SUVmean/Liver SUVmean.

Of the 30 patients who underwent baseline and interim^18^F-FDG PET/CT scans,16 (53.3%) presented an increase ≥30% (Group ≥ 30%) in^18^F-FDG LV SUVmax. Regarding clinical variables, such as cardiovascular risk factors and drugs in use, no differences were observed.

The values of the LV SUVmax/aorta SUVmax and LV SUVmean/liver SUVmean ratios also increased significantly at the interim evaluation compared to the baseline in the group ≥30% ( Figure 1B ). In the group<30% (n=14), there was no statistically significant increase in these ratios from baseline to interim scans ( Figure 1C ).

### Group 2: baseline and post-therapy 18F-FDG PET/CT

Sixty-six patients underwent baseline and post-therapy^18^F-FDG PET/CT scans. No statistically significant differences were seen in FBS,^18^F-FDG injected activity and time post-injection were found between the two evaluations. Patients’ mean body weight was slightly higher in the post-therapy scan compared with baseline ( Table 3 ).

**Table 3:** – Comparison of body weight, fasting blood sugar, injected dose of 18F-fluorodeoxy-glucose (18FDG), and mean post-injection timel of patients between baseline and post-therapy positron emission tomography associated with computed tomography scans (PET/CT)

Variable (N=66)	Baseline Pet	Post-Therapy Pet	p*
	Mean ± SD	Mean ± SD	
**Weight** (Kg)	72.7 ± 14.8	75.2 ± 15.2	0.014
**FBS** (mg/dL)	91.6 ± 15.6	91.6 ± 16.7	>0.99
**Dose of** ^18^FDG mCi	9.2 ± 2.3	9.5 ± 2.2	0.308
**Post-injection time** (min)	68.6 ± 9.1	70.4 ± 5.8	0.606

*Student’s t-test. FBS: Fasting Blood Sugar

The mean value of the LV SUVmax was significantly higher in the post-therapy PET. We observed an absolute increase in the^18^F-FDG cardiac uptake value of 2.1 (95% CI:1.3 to 3.0), which represents a percentage increase of 66.5% (95%CI:43.3% to 89.7%) over the baseline scan.

The values of the LV SUV max/aorta SUV max and the LV SUV mean/liver SUV mean ratios also increased significantly in the post-therapy PET as compared with baseline, Figure 2A . The mean time between baseline and post-therapy exams was 231.8±125.7 days.

**Figure 2:** – Group 02 – A) Comparison of LV SUVmax, LV SUVmax/Aorta SUVmax and LV SUVmean/Liver SUVmean ratios, between Baseline and Post-therapy PET. B) Comparison of LV SUVmax/Aorta SUVmax and LV SUVmean/Liver SUVmean ratios, between Baseline and Post-therapy PET in the Group with increase of LV SUVmax ≥ 30%. C) Comparison of LV SUVmax/Aorta SUVmax and LV SUVmean/Liver SUVmean ratios, between Baseline and Post-therapy PET in the Group with increase of LV SUVmax < 30%; LVmaxAOmax: LV SUVmax/Aorta SUVmax, LVmean LIVER mean: LV SUVmean/Liver SUVmean.

Of the 66 patients, 38 (57.6%) presented ≥30% increase in^18^F-FDG cardiac uptake (Group ≥ 30%). There were no differences between the groups regarding the clinical variables, such as cardiovascular risk factors and medications in use.

The values of the LV SUVmax/aorta SUVmax and LV SUVmean/liver SUVmean ratios increased significantly in the post-therapy evaluation compared to the baseline in the ≥30% group ( Figure 2B ). In the Group<30% (n=28), there was no statistically significant increase in the ratios ( Figure 2C ).

Figure 3 illustrates a case example of the^18^F-FDG LV SUV max behavior before, during and after chemotherapy.

**Figure 3:** – Case example - LV SUVmax in Baseline (5.86), Interim (8.95 / 52.73% percentage increase from baseline) and Post-therapy PET/CT (9.67 / 65.02% percentage increase from baseline). LV: Left Ventricle; PET/CT: Positron emission tomography associated with computed tomography scans; SUV: Standard Uptake Value; SUVmax: Maximum SUV.

## Discussion

The present study showed that chemotherapy in patients with lymphoma caused an unbalance in cardiac metabolism, evidenced by a higher myocardial^18^F-FDG uptake. These results are supported by recent evidence suggesting that it may be an early sign of CTX in response to the redox stress. The cardiac^18^F-FDG increase occurred in more than 50% of the patients and was observed in the interim PET and in the post-therapy scan. These results suffered no interference regarding the^18^F-FDG injected activity or any possible differences in exam preparation and timing.

The^18^F-FDG PET/CT is a well-established method in the diagnosis and staging of oncologic patients, especially with lymphoma, with a potential capacity to assess early manifestations of CTX in a way analogue to the ischemic cascade, as postulated in Figure 4 .

**Figure 4:** – Cardiotoxicity cascade – Cardiotoxic injury triggers series of metabolic alterations in response to the oxidative stress, it is detectable by 18F-FDG PET/CT. The sustained injury and the failure of the myocyte self-healing contribute to cell dysfunction and mechanic alterations detected by strain rate imaging. Furthermore, the process continues with a decrease in the cardiac overall performance assessed by the LVEF. Signs of heart failure are then noticeable, suggesting that the heart no longer meet the body’s demands, or do it at the expense of high ventricular filling pressures (ROS: reactive oxygen species; ER: endoplasmic reticulum; PPP: pentose phosphate pathway; H6PD: hexose-6-phosphate dehydrogenase; FDG: 18F-fluorodeoxy-glucose; LVEF: Left Ventricle Ejection Fraction).

Antineoplastic therapies have improved overall survival rates in oncologic patients. However, their cytotoxic effects have shown a wide spectrum of acute and chronic alterations to the cardiovascular system.^19^ The cellular and molecular mechanisms of CTX are known to disrupt the redox homeostasis mostly in the myocardium and endothelium, significantly impairing cardiovascular health.^20^

CTX affects the cardiovascular system first by the inhibition of topoisomerase II and the formation of ROS. The intrinsic mitochondria-dependent and extrinsic death receptor pathways of apoptosis are then triggered. The cascade continues with the activation of caspase^3^ , phosphatidylserine expression, DNA fragmentation, chromatin condensation, and phospholipid membrane metabolization.^21^ The final stage is characterized by membrane blebbing and cell shrinkage.^22^ This is the mechanism underlying subclinical CTX and it provides various opportunities to assess early signs of this entity.

The current recommendations and guidelines rely on imaging techniques focused on anatomy-based parameters, such as echocardiography, multigated radionuclide angiography (MUGA), and cardiac magnetic resonance imaging (CMRI).^23^ However, these approaches detect late manifestations of CTX with low sensitivity for subclinical alterations.^24^

Nuclear medicine techniques may be a tool to assess specific points of the CTX pathway. The^18^F-FDG PET/CT, commonly used to detect tumoral glycolytic metabolism, has presented itself as an early marker of CTX. Initially, several studies pointed out that doxorubicin (DXR), one of the most utilized anthracyclines, can specifically affect myocardial metabolism, as showed by experimental study.^25^

Several experimental and clinical studies have shown that cardiotoxic therapy, such as sunitinib and anthracyclines, increases the cardiac^18^F-FDG uptake over time and is related to echocardiographic alterations.^26 - 33^

Although^18^F-FDG uptake has been commonly associated with glucose consumption, more recent data have shown otherwise. The redox stress and its antioxidant response have been characterized as a possible mechanism behind the progression of cardiac contractile impairment in CTX and in the^18^F-FDG uptake independently of the glycolytic metabolism.^34^

Redox stress to the endoplasmic reticulum (ER) environment might activate the local H6PD-triggered pentose phosphate pathway to fuel the NADPH levels needed for the antioxidant response, and is related to an increased^18^F-FDG uptake.^35^

In situations of oxidative stress, NADPH is a major source of electrons for reductive reactions.^36^ It is generated intraluminally by H6PD, a bifunctional enzyme that catalyzes the first two steps of the pentose phosphate pathway, converting glucose-6-phosphate to 6-phosphogluconate with the concomitant production of NADPH.^37^ H6PD has as substrate several hexoses such as 2-deoxyglucose and FDG.^38^

In the heart, there is a direct link between ER oxidative stress and myocardial uptake of 2-deoxyglucose,^39^ that may be considered an early metabolic phase of contractile dysfunction by pressure overload.^40^ Furthermore, Hrelia et al.^41^ showed that the increase of 2-deoxyglucose uptake induced by DXR in cardiomyocytes can be reverted by the antioxidant effect of alpha-tocopherol.^41^

Bauckneht et al.,^33^ in 2019, analyzed the effect of DXR -induced oxidative damage on the correlation between myocardial^18^F-FDG uptake, overall glucose consumption and the H6PD-triggered metabolic response in mice. The study showed that myocardial redox stress persisted and directly correlated with the enhancement in^18^F-FDG uptake (SUV increase), and the activation of physiological antioxidant pathways such as the catalytic function of H6PD.^33^ The study also showed that the metabolic alteration persisted after the disappearance of DXR, and it preceded the manifestation of contractile impairment.^33^ Previous reports also showed a positive loop connecting ROS generation and^18^F-FDG uptake in cancer.^42^

In agreement with these findings, recent studies showed an increased^18^F-FDG uptake on PET/CT independent of glycolytic metabolism and linked to the enzymatic activity of H6PD in the brain.^43 , 44^ Another analysis showed the link between^18^F-FDG uptake and ROS generation in hyperglycemia-induced redox stress involving H6PD activation.^45^

Despite its interesting results and background of the present study, its retrospective nature makes the assessment of the mechanisms underlying the increased myocardial^18^F-FDG uptake difficult. However, no other cardiotoxic factors, besides CTX, were identified between baseline and control exams in the largest sample of patients with lymphoma evaluated during and after chemotherapy. In addition, unlike the other studies, we measured not only the LV SUVmax, but also the LV uptake values corrected for liver and blood pool, as control, confirming the increase of the cardiac uptake. Furthermore, the^18^F-FDG PET/CT protocol and the possible factors of SUV variability were the same in all baseline and control scans.

More studies are necessary to correlate increased cardiac^18^F-FDG uptake with clinical outcomes, the class and dose of chemotherapy, troponin and NT-proBNP levels, and with other imaging methods such as echocardiography and CMRI.

## Conclusion

The present study showed a clear increase in cardiac^18^F-FDG uptake in patients with lymphoma, verified by^18^F-FDG PET/CT during and/or after chemotherapy. The literature corroborates with these findings and suggests that it may be an important and early sign of CTX that can be easily assessed by a widely available method. With the progressive improvement in anticancer therapies, CTX is still a concern that requires further investigation and new diagnostic approaches.
